# Characteristics and antioxidant activity of Maillard reaction products from β-lactoglobulin and isomaltooligosaccharide

**DOI:** 10.3389/fnut.2023.1282485

**Published:** 2023-10-17

**Authors:** Qingyu Wang, Jiayang Li, Yaqi Tu, Jianping Cai, Fazheng Ren, Hao Zhang

**Affiliations:** ^1^The Key Laboratory of Geriatrics, Beijing Institute of Geriatrics, Institute of Geriatric Medicine, Chinese Academy of Medical Sciences, Beijing Hospital/National Center of Gerontology of National Health Commission, Beijing, China; ^2^College of Food Science and Nutritional Engineering, China Agricultural University, Beijing, China; ^3^Beijing Laboratory of Food Quality and Safety, Department of Nutrition and Health, China Agricultural University, Beijing, China; ^4^Food Laboratory of Zhongyuan, Luohe, Henan, China

**Keywords:** Maillard reaction products, antioxidant activity, β-lactoglobulin, isomaltooligosaccharide, dairy protein

## Abstract

Starch-derived isomaltooligosaccharide (IMO) is potentially used as prebiotics in infant formulas. Given that they are non-digestible carbohydrates rich in reducing substrates, it’s crucial to understand if they can interact with β-lactoglobulin (β-LG) to produce Maillard reaction products (MRPs) and how these MRPs might influence the nutritional properties of β-LG. In our investigation, we conjugated β-LG with IMO to generate MRPs. Using a spectrophotometer, we identified the intermediates and assessed browning. We also evaluated changes in free amino groups and structural alterations. The antioxidative activity of the resulting compounds was assessed using DPPH and the ferric reducing/antioxidant power (FRAP) assay. Our data revealed increased visible absorption and fluorescence intensity, suggesting the formation of intermediate and browning products. The content of free amino groups diminished by 33%, supporting the conjugation of IMO with β-LG. However, circular dichroism results indicated no significant alterations in the secondary structure of β-LG. Notably, the β-LG-IMO MRPs exhibited enhanced 2,2-diphenyl-1-picrylhydrazyl (DPPH) radical-scavenging activity and ferric reducing/antioxidant power (FRAP). The findings provide insights into the characteristics and antioxidant activities of the conjugates derived from IMO and dairy protein in infant formula.

## 1. Introduction

The Maillard reaction occurs when reducing sugar carbonyl groups react with nitrogenous amino groups. This reaction is especially significant during the processing or extended storage of dairy products (1, 2). Given the presence of reducing sugars and amino acids, dairy products are particularly prone to the Maillard reaction (3). Heat treatment, commonly employed in the dairy industry for sterilization and drying, further facilitates this reaction (4). As a functional ingredients, Maillard reaction products (MRPs) contributes to the color, flavor, and bioactivity of foods (5). Beyond these attributes, MRPs also enhance gelling, emulsifying, and provide anti-inflammatory, antibacterial, bifidogenic, and antioxidant properties to proteins (6). Given these benefits, the Maillard reaction is widely used as a pivotal strategy to improve the functional properties of food ingredients.

β-lactoglobulin (β-LG) constitutes 40–50% of the total whey protein in bovine milk, offering significant nutritional values and versatility in food processing (7). Several studies have investigated the Maillard reaction between various sugars and β-LG in infant formulas (8), reporting that MRPs derived from different sugars exhibit diverse properties. It has been observed that the β-LG-tagatose (aldose) reaction system presents a higher fluorescence intensity compared to the β-LG-lactose (ketose) system, underscoring the influence of the carbonyl group in reducing sugars during Maillard reaction’s progression (9). Additionally, the higher antioxidant capacity of the β-LG-galactose reaction system had a higher antioxidant capacity than the β-LG-lactose reaction system, reinforces the correlation between the antioxidant activity of MRPs and the type of the reducing sugars (10).

Isomaltooligosaccharide (IMO) consists of glucose oligomers linked by α-(1 → 6) bonds, encompassing carbohydrates such as isomaltose, panose, isomaltotriose, isomaltotetraose, and isomaltopentaose, with degrees of polymerization ranging from 2 to 10 (11). IMO has already been incorporated as a prebiotic in infant formula (12). Evidences has shown its ability to improve probiotic proliferation in the intestine and modulate the intestinal microbiota homeostasis (13, 14). Although IMO has already been applied in dairy products, as carbohydrates, limited studies have explored the characteristics and antioxidative properties of MRPs derived from the interaction of IMO and dairy proteins.

The aim of this study was to determine the MRPs derived from the interaction of β-LG and IMO, and to identify their characteristics and antioxidant properties. Throughout various production stages, we used UV–visible absorbance along with fluorescence intensity to characterize the MRPs. Alterations in the content of free amino groups provided insights into the extent of the Maillard reaction. Circular dichroism (CD) was performed to identify the secondary structural changes of β-LG. Additionally, the 2,2-diphenyl-1-picrylhydrazyl (DPPH) radical scavenging activity and ferric reducing/antioxidant power (FRAP) assays were used to assess the antioxidant properties of the MRPs.

## 2. Materials and methods

### 2.1. Chemicals and reagents

β-LG (L3908, purity ≥90%), 2,4,6-tris(2-pyridyl)-s-triazine (TPTZ) (purity ≥98%), and DPPH were obtained from Sigma Aldrich Chemical Co. (St. Louis, MO, United States). IMO (food grade, the degree of polymerization of 2–10) was obtained from Shandong Bailong Chuangyuan Biotechnology Co., Ltd. (DeZhou, Shandong, China). O-Phthalaldehyde (OPA) (purity 98%) was obtained from Tianjin Jinke Fine Chemical Research Institute (Tianjin, China). Sodium dodecyl sulfate (SDS) and glycine (purity >99%) were obtained from Solarbio Co., Ltd. (Beijing, China). Sodium tetraborate decahydrate (purity ≥99.5%) was obtained from Beijing Chemical Industry Group Co., Ltd. (Beijing, China). β-Mercaptoethanol (purity ≥99%) was obtained from Kulaibo Technology Co., Ltd. (Beijing, China). The protein molecular weight marker was obtained from Beyotime Biotechnology (Shanghai, China). Ascorbic acid (purity ≥99.7%) was obtained from Beijing Aoboxing Biotechnology Co., Ltd. (Beijing, China). All other reagents (analytical grade) were obtained from Tianjin Yongda Chemical Reagent Co., Ltd. (Tianjin, China).

### 2.2. Preparation of β-LG-IMO conjugates

The conjugates of β-LG-IMO were prepared based on a previously described method with minor modifications (15). A solution of 5% (w/v) β-LG and 10% (w/v) IMO was prepared in distilled water and allowed to dissolve overnight at 4°C, followed by lyophilization. The mixture was incubated at a steady temperature of 60°C for 0, 12, 24, 48, and 72 h in a dryer containing saturated potassium bromide solution (relative humidity of 79%). As references, both β-LG and IMO were also incubated individually. Each treatment was independently prepared three times.

### 2.3. Determination of absorbance and fluorescence intensity

The development of intermediates and browning in MRPs was measured at absorbances of 294 nm and 420 nm, respectively (16). Samples at a concentration of 2 mg mL^−1^ (dissolved in Milli-Q water) were determined at an absorbance of 294 nm to analyze the intermediates. Samples at a concentration of 16 mg mL^−1^ (dissolved in Milli-Q water) were determined at an absorbance of 420 nm to evaluate the browning intensity. Milli-Q water served as the blank for absorbance measurements.

The fluorescence intensity of the prepared sample solutions was assessed at 350/420 nm (excitation/emission) according to previous literatures (17, 18). Fluorescent substances, which form prior to the appearance of brown pigments, have been used to determine the progression of the Maillard reaction (19). All samples were adjusted to a protein concentration of 0.5 mg mL^−1^ with Milli-Q water before assessment. Milli-Q water was used to determine the blank absorbance value. All fluorometric experiments were conducted using a multimode reader (Infinite M200 Pro, Tecan, Switzerland).

### 2.4. Measurement of the content of free amino groups

The content of free amino groups was determined by the previous method with sightly-modified (20). A fresh *o*-phthalaldehyde (OPA) reagent (50 mL) was prepared using the following reagents mixed with Milli-Q water: 40 mg mL^−1^ OPA (dissolved in methanol), 100 μL β-mercaptoethanol, 2.5 mL SDS solution (20% w/v) and 25 mL sodium borate buffer (100 mM, pH = 9.5). The OPA reagent (200 μL) was mixed with a 20 μL sample containing 6 mg mL^−1^ protein. The absorbance value of the solution was detected at 340 nm after the reaction was maintained at 25°C for 5 min. The quantity of free amino groups was determined based on a glycine standard curve (0–20 mmol L^−1^). The final values were presented as the percentage (%) content of free amino groups relative to the control.

### 2.5. Circular dichroism measurements

The CD spectra of the β-LG-IMO samples were measured by a CD spectropolarimeter (Pistar π−180; Applied Photophysics Ltd., Leatherhead, United Kingdom) in the far ultraviolet range of 190 nm to 260 nm. A 200 μL sample (diluted in Milli-Q water at a protein concentration of 0.2 mg mL^−1^) was loaded into a 1 mm quartz cuvette. The spectra were acquired at a scan speed of 120 nm/min with a 1 nm bandwidth. Solvent background spectra were subtracted to correct the sample spectra.

### 2.6. DPPH radical scavenging activity

The DPPH radical scavenging activity was determined according to the previous method with slight modification (21). A mixture of 120 μL of DPPH in ethanol (0.1 mM) and 60 μL of the sample with a protein concentration of 16 mg mL^−1^ protein was prepared and allowed to react in the dark for 30 min at 25°C. The mixture was then centrifuged at 1500 × g for 2 min. The absorbance of the resulting supernatant was measured at 517 nm. A control sample was treated similarly for comparison. The scavenging activity was computed according to Eq. (1):


(1) $$\begin{array}{l} \mathrm{DPPH}\;\mathrm{radical}\;\mathrm{scavenging}\;\mathrm{activity}\;\left(\%\right)\\ \;=\left(1-A_{517\;\mathrm{nm}\;\mathrm{sample}}/A_{517\;\mathrm{nm}\;\mathrm{control}}\right)\times 100 \end{array}$$

where: *A_517 nm sample_* and *A_517 nm control_* are the absorbance at 517 nm of the sample and the control, respectively. The IC_50_ value represents the sample concentration required to achieve 50% DPPH radical scavenging activity.

### 2.7. Ferric reducing/antioxidant power

The FRAP values of MRPs were measured according to the previous study (22). The FRAP reagent was prepared by combining 1 mL of TPTZ solution (10 mM), 1 mL of FeCl_3_ solution (20 mM), and 10 mL of acetate buffer (300 mM, pH 3.6). The FRAP reagent (160 μL) was mixed with 20 μL of the sample containing 16 mg mL^−1^ protein. After incubating at 37°C for 30 min, the absorbance of the mixture was measured at 595 nm. Blank samples were prepared similarly. Ascorbic acid solutions ranging from 0–1,000 μM were used to establish the calibration curve. The outcomes were expressed in terms of μM equivalents of ascorbic acid per gram of protein.

### 2.8. Statistical analysis

All experiments were conducted in triplicates and values were expressed as mean ± standard deviation. SPSS 25.0 software and Origin 2018 were used for data analysis and image processing, respectively. The statistical significance between mean values was analyzed by one-way analysis of variance (ANOVA) followed by post-hoc Tukey’s test. *p* < 0.05 was considered statistically significant (23).

## 3. Results and discussion

### 3.1. The MRPs produced by IMO and β-LG conjugation

**Figure 1:**
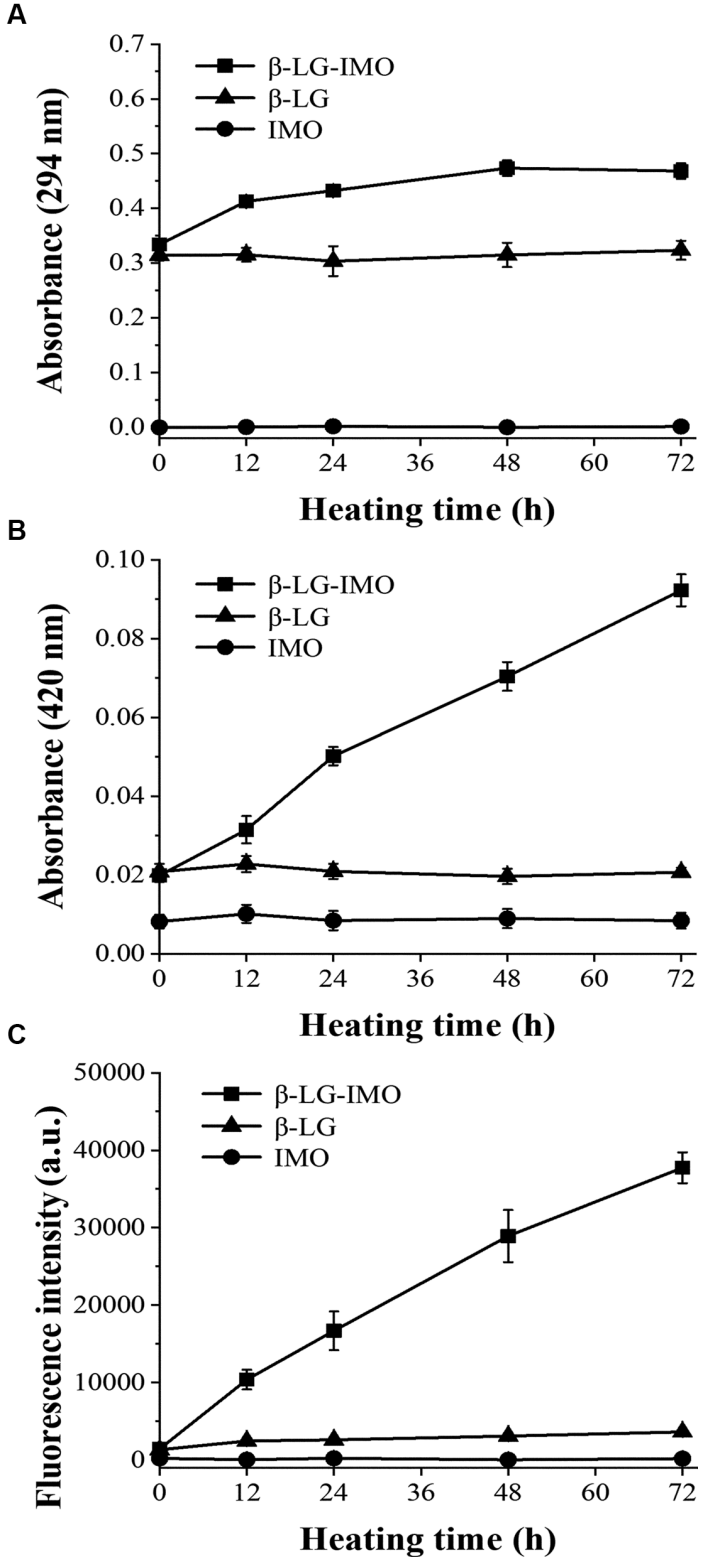
Variations in the absorbance at wavelengths of 294 nm **(A)** and 420 nm **(B)**, and fluorescence intensity at 350/420 nm (excitation/emission) **(C)** for β-lactoglobulin-Isomaltooligosaccharide (β-LG-IMO), β-lactoglobulin (β-LG), and isomaltooligosaccharide (IMO) post-heat treatment at 60°C and 79% relative humidity for a duration of 72 h. Data represent the mean of three independent experiments.

The production amounts of intermediate products and brown substances can be evaluated by the Ultraviolet–visible absorbance at 294 and 420 nm, respectively. As shown in Figure 1A, after 12 h of incubation at 60°C, MRPs samples exhibited a significant (*p* < 0.05) increase in absorbances at 294 nm and reach the plateau at 48 h, indicating that the great majority of intermediate products were produced within the first 48 h, with no notable changes observed when the heating duration was extended to 72 h. This trend is likely attributed to the polymerization of intermediates into melanoproteins as the heating period extends (24).

Figures 1B,C demonstrate that as the reaction time extended, the absorbance at 420 nm and the fluorescence intensity of the β-LG-IMO reaction system increased significantly (*p* < 0.05). No notable changes (*p* > 0.05) were observed when either β-LG or IMO was heated independently. This indicates that the formation of brown compounds and fluorescent substances predominantly occurs when β-LG and IMO are heated in conjugation. The results from an earlier report suggested that the aldol and aldehyde-amine condensations of reductones, fission, and Strecker degradation products play a part in making the brown nitrogenous compounds and macromolecular substances at the advanced stage of the reaction (25). The observed fluorescence arises from the interaction between reductive substances and the Strecker degradation products of amines and intermediates (26, 27). In addition, during the intermediate and advanced stages of Maillard reaction, a series of complex reactions lead to the breakdown of Amadori products and the formation of carbonyl compounds. These compounds can rearrange, cyclize, and polymerize, giving rise to advanced glycation end products (AGEs). AGEs are complex and heterogeneous compounds known for their brown color and fluorescent cross-linking substances such as pentosidine (28, 29). As the concentration of AGEs increases during the progression of the Maillard reaction, fluorescence intensity correspondingly rises. Thus, monitoring this fluorescence can indicate the Maillard reaction progress.

### 3.2. Changes in the content of free amino groups

**Figure 2:**
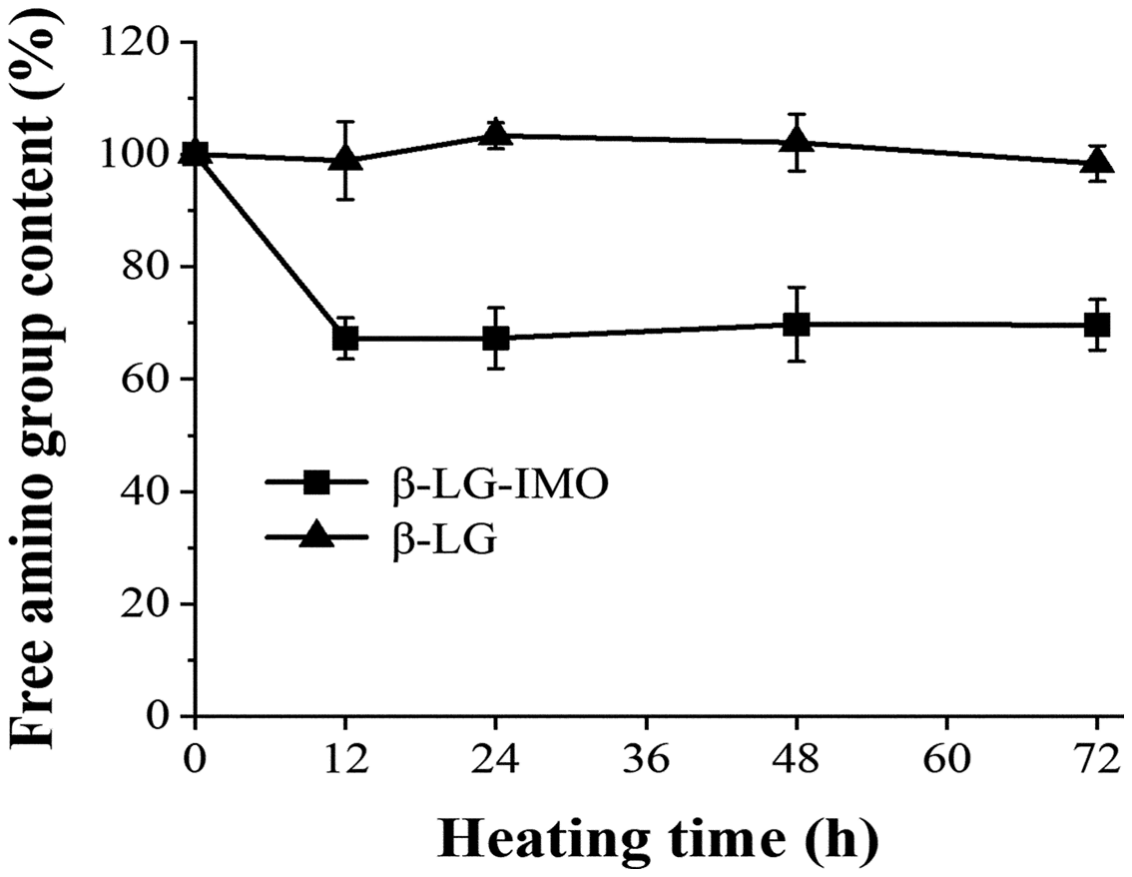
Alterations in the free amino group content of β-lactoglobulin-isomaltooligosaccharide (β-LG-IMO) and β-lactoglobulin (β-LG) under heat exposure at 60°C and 79% relative humidity for a duration of 72 h. Data represent the mean of three independent experiments.

Figure 2 shows the amount of free amino groups in β-LG when heated for up to 72 h, both in the presence and absence of IMO (control group). There was no significant change (*p* > 0.05) in the contents of free amino groups when β-LG was heated alone. In contrast, when β-LG was heated in conjunction with IMO for 12 h, there was a substantial reduction, by roughly 33%, in the content of free amino groups. This decline in the free amino group content was not due to the release of NH2 groups from the β-LG substrate. Instead, it resulted from the interaction between sugar and free amino acids during the Maillard reaction (30), and lysine is reported to be the primary amino acid that is consumed during the reaction (31). However, as the reaction progressed, the loss of free amino groups remained relatively constant which was consistent with the finding of prior research (32). This stabilization can likely be attributed to the rapid binding of free amino groups, such as ε-NH_2_ of lysine residues and α-amino groups present at the N-terminus of proteins, with IMO in the initial 12 h (33, 34).

### 3.3. The structural detection of MRPs

**Figure 3:**
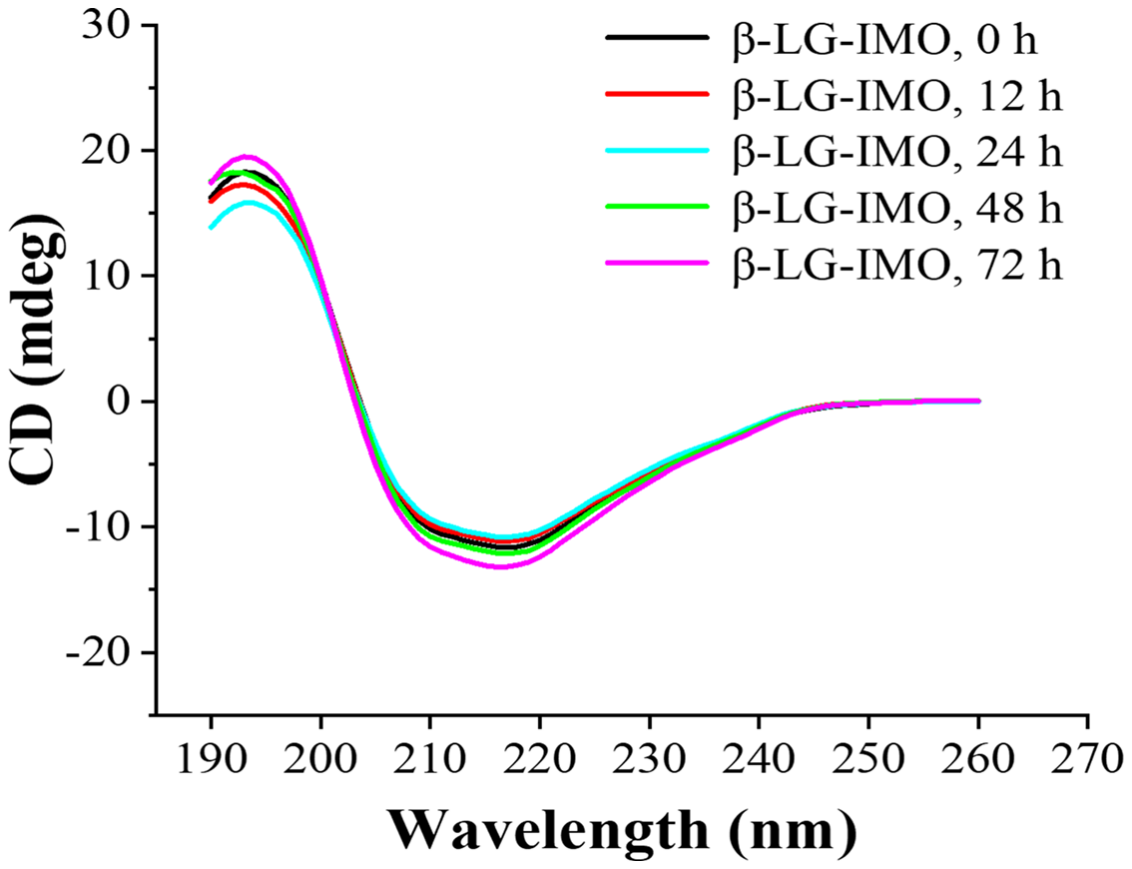
Far-UV CD spectra of β-lactoglobulin-isomaltooligosaccharide (β-LG-IMO) subjected to heat treatment at 60°C and 79% relative humidity for a duration of 72 h. Each spectrum is an average obtained from three independent measurements.

Far-UV CD is a technique used to identify alterations in the secondary structure of proteins (35). The obtained CD spectra and the composition of the secondary structure are presented in Figure 3 and Supplementary Table S1, respectively. A positive peak observed around 203 nm indicated the existence of the α-helical structure. A broad negative peak distributed between 210 to 220 nm implies a high β-sheet content in the β-LG. This is consistent with findings which state that β-LG predominantly has a β-sheet structure, consisting of 9 anti-parallel β-strands and a major α-helix situated at the C-terminus of the molecule (36). The untreated β-LG-IMO exhibited levels of 22.25% α-helix, 25.49% β-sheet, 16.46% β-turn, and 35.79% random coil. However, with the continuation of the heating process through 12, 24, 48, and up to 72 h, no further changes (*p* > 0.05) in the secondary structural components of β-LG were detected across all the treatments. This observation aligns with prior findings (37), suggesting that such a phenomenon might be associated with the reactivity between sugar and its binding sites on the protein.

### 3.4. DPPH radical scavenging activity

**Figure 4:**
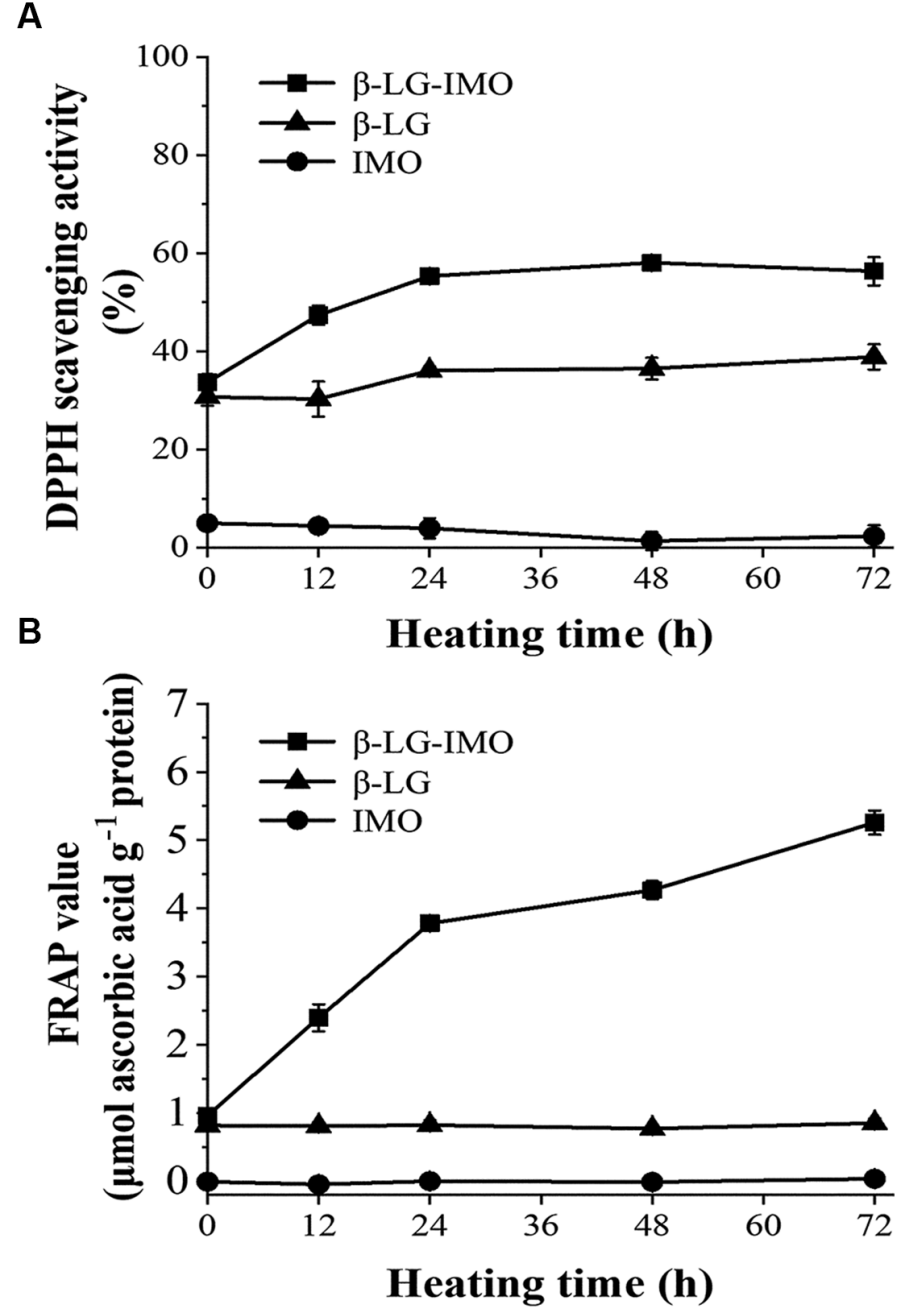
Variations in the DPPH radical scavenging activity **(A)** and ferric reducing/antioxidant power (FRAP) **(B)** of β-lactoglobulin-isomaltooligosaccharide (β-LG-IMO), β-lactoglobulin (β-LG), and isomaltooligosaccharide (IMO) upon heat exposure at 60°C and 79% relative humidity for 72 h. Data represent the mean of three independent experiments.

DPPH assays are commonly used to determine the antioxidant activity of a variety of food materials (38). Antioxidants can scavenge the DPPH radical via donating hydrogen, resulting in the formation of a stable DPPH-H molecule, which can be spectrophotometrically measured at 517 nm (39). Figure 4A shows that IMO demonstrated no significant DPPH radical scavenging capacity, while the β-LG-IMO MRPs exhibited significantly higher DPPH radical-scavenging activity than the controls. The IC_50_ value of β-LG-IMO conjugates was 14.5 mg mL^−1^, which was significantly lower than that of β-LG or IMO itself (neither reached IC_50_ at their maximum solubility). The DPPH radical scavenging activity of β-LG was approximately 33%, indicating its antioxidant properties. This antioxidative potential of β-LG could be attributed to its free cysteine-121 residue, whose sulfhydryl group can donate hydrogen to neutralize free radicals (40). Additionally, the tryptophan residue in β-LG is also regarded as an effective free radical scavenger (41). MRPs have been found to scavenge DPPH radicals mainly due to the production of melanoidins, which are capable of scavenging reactive oxygen species, capturing free radicals, and chelating metals at the final stages of the Maillard reaction (42). The above results suggest that MRPs provide a protective effect against oxidative damage.

### 3.5. FRAP

In Figure 4B, the FRAP values of the control groups, when heated individually, displayed minimal variations throughout the entire heating duration (*p* > 0.05). In contrast, with an extended heating duration, the FRAP values of the MRPs derived from the interaction of β-LG and IMO increased remarkably (*p* < 0.05), which correlates with the findings from the DPPH radical scavenging assay. These observations indicated that hydroxyl and pyrrole groups present in MRPs might act as electron donors, facilitating the reduction of Fe^3+^ to Fe^2+^ (43). In addition, the reducing ketones and brown compounds produced in the Maillard reaction could also attribute to antioxidant activity as electron donors (44).

## 4. Conclusion

In this study, we investigated the characteristics and antioxidant properties of MRPs derived from the reaction between β-LG and IMO. Using spectrometric analysis and free amino group measurement, we identified the formation of browning and intermediate products resulting from the Maillard reaction. The secondary structure of the dairy protein remained relatively stable after the Maillard reaction. The most significant finding are that MRPs generated from β-LG-IMO conjugation demonstrated robust DPPH radical-scavenging activity (approximately twice that of β-LG) and high FRAP values (with a maximum value approximately five times that of β-LG), suggesting that these MRPs provide a protective effect against oxidative damage. A limitation of our study is that it is based on a simulated system, and the changes in actual infant formula remain to be investigated. Additionally, further *in vivo* investigations are needed to validate the functional properties. Overall, our findings highlight the potential of MRPs derived from IMO and β-LG to enhance infant formula and underscore their ROS scavenging capacities, which augments the functional benefits of IMO.

## Data availability statement

The raw data supporting the conclusions of this article will be made available by the authors, without undue reservation.

## Author contributions

QW: Conceptualization, Data curation, Formal analysis, Software, Visualization, Writing – original draft. JL: Methodology, Software, Validation, Writing – review & editing. YT: Methodology, Software, Validation, Writing – review & editing. JC: Methodology, Software, Validation, Writing – review & editing. FR: Investigation, Resources, Writing – review & editing. HZ: Funding acquisition, Investigation, Project administration, Supervision, Writing – review & editing.
