# Supplementary material for: Characteristics and antioxidant activity of Maillard reaction products from β-lactoglobulin and isomaltooligosaccharide
Source: Front Nutr. 2023 Oct 17;10:1282485. doi: 10.3389/fnut.2023.1282485 (PMC10616461; doi:10.3389/fnut.2023.1282485)
Supplement: Supplementary file 1 [file Table_1.DOC]

Table S1

Changes in the secondary structure content of β-lactoglobulin-isomaltooligosaccharide (β-LG-IMO).^a^

| Samples | α-Helix (%) | β-Sheet (%) | β-Turn (%) | Random coil (%) |
| --- | --- | --- | --- | --- |
| β-LG-IMO, 0 h | 22.25 ± 3.26^a^ | 25.49 ± 3.93^a^ | 16.46 ± 0.10^a^ | 35.79 ± 0.57^a^ |
| β-LG-IMO, 12 h | 21.60 ± 1.22^a^ | 26.22 ± 1.49^a^ | 16.45 ± 0.06^a^ | 35.73 ± 0.21^a^ |
| β-LG-IMO, 24 h | 20.92 ± 0.39^a^ | 27.03 ± 0.60^a^ | 16.49 ± 0.03^a^ | 35.56 ± 0.21^a^ |
| β-LG-IMO, 48 h | 22.59 ± 2.79^a^ | 25.32 ± 3.16^a^ | 16.55 ± 0.10^a^ | 35.54 ± 0.31^a^ |
| β-LG-IMO, 72 h | 23.63 ± 1.82^a^ | 24.28 ± 1.79^a^ | 16.66 ± 0.07^a^ | 35.42 ± 0.27^a^ |

^a^ The data are shown as mean ± standard deviation (n = 3). Same letter superscripts in the same column indicate no significant difference (*P* > 0.05). Samples were heated at 60 °C, 79% relative humidity for up to 72 h.
